# Gated Graph Attention Network for Cancer Prediction

**DOI:** 10.3390/s21061938

**Published:** 2021-03-10

**Authors:** Linling Qiu, Han Li, Meihong Wang, Xiaoli Wang

**Affiliations:** School of Informatics, Xiamen University, Xiamen 361001, China; qiulinling@stu.xmu.edu.cn (L.Q.); lihan@stu.xmu.edu.cn (H.L.); xlwang@xmu.edu.cn (X.W.)

**Keywords:** attention mechanism, gating mechanism, graph convolutional network, TCGA, cancer prediction

## Abstract

With its increasing incidence, cancer has become one of the main causes of worldwide mortality. In this work, we mainly propose a novel attention-based neural network model named Gated Graph ATtention network (GGAT) for cancer prediction, where a gating mechanism (GM) is introduced to work with the attention mechanism (AM), to break through the previous work’s limitation of 1-hop neighbourhood reasoning. In this way, our GGAT is capable of fully mining the potential correlation between related samples, helping for improving the cancer prediction accuracy. Additionally, to simplify the datasets, we propose a hybrid feature selection algorithm to strictly select gene features, which significantly reduces training time without affecting prediction accuracy. To the best of our knowledge, our proposed GGAT achieves the state-of-the-art results in cancer prediction task on LIHC, LUAD, KIRC compared to other traditional machine learning methods and neural network models, and improves the accuracy by 1% to 2% on Cora dataset, compared to the state-of-the-art graph neural network methods.

## 1. Introduction

The incidence of cancer has increased year by year. According to the World Health Organization (WHO)’s latest report *Global Cancer Statistics 2018* [1], there were 18.1 million new cancer cases (9.5 million men and 8.6 million women), and 9.6 million deaths (5.4 million men and 4.2 million women) in the year of 2018, further aggravating the global economic and medical burdens [2]. In addition, the WHO also pointed out that one third of cancer cases are completely preventable [3]. Therefore, early accurate cancer prediction and evaluation in high-risk groups (people aged 50–60, overeating, smoking, etc.) is very important to reduce cancer-related pain and mortality. It is widely known that early detection can minimize the risk of cancer cell proliferation and ensure appropriate treatment at the beginning of cancer, which can also greatly reduce the social medical costs and family economic burden.

The earliest cancer prediction is based on gene sequencing technology, such as Sanger sequencing technology and high-throughput sequencing technology, which is used for gene detection in the molecular level. This technology uses some physical testing instruments (like amplification instrument, sampling instrument, thermostatic equipment for chip hybridization, scanner for reading chip results) for gene localization, to detect and discover specific gene mutations related to diseases using DNA hybridization and other principles. However, the cost of using the testing instruments is high, the operation is complicated, and the degree of automation is low, which may not meet the needs of large-scale gene sequencing [4].

The data driven methods (like machine learning methods and deep learning methods) omits the complex machine operation steps, and carries out the gene related tasks through the algorithms by training the models on the datasets, which is simple, efficient, accurate, and automatic.

Some traditional machine learning methods, such as Naive Bayes (NB), K-nearest Neighbor (KNN), Decision Tree (DT), Support Vector Machine (SVM), Random Forest (RF), etc., have also achieved good results in cancer prediction [5,6,7,8,9,10,11,12,13,14,15,16,17,18,19]. But these methods usually lack the correlation analysis between each sample, which fails to extract practical features and may lead to large calculations (We notice that batching calculations is a widely-used approach to reduce the amount of calculation. Many implementations utilise random sampling to create batches, which narrow the samples down to a smaller range in training. However, we have to admit that: (1) batching does not make much sense unless the training samples are large in size; and (2) batching destroys the corelations, or relations, between samples, which are vital to our method proposed in this article) [20], that is, they treat all features as a whole without any selection, which is inefficient. It is possible that these methods may achieve lower accuracy in predicting newly-discovered disease than it has performed in traditional datasets, because the underlying information is not mined thoroughly. In recent years, deep learning methods achieve higher notice in cancer prediction based on the strong feature learning ability of the neural networks, which may achieve high accuracy in cancer prediction as well as other fields [21,22,23,24,25,26,27,28,29,30,31].

Machine learning methods are in general employed to deal with two kinds of data: Euclidean and non-Euclidean structured data. Recently, the study of deep learning methods on graph has made some achievements on non-Euclidean structured data. A Euclidean dataset is sensibly modelled as being plotted in an *n*-dimensional linear space. For example, image files are considered a typical sub-class of Euclidean data, where the *x* and *y* coordinates refer to the location of each pixel and the *z* coordinate signifies its colour/intensity. On the contrary, a non-Euclidean dataset is not constructed regularly and usually in graph-structured shapes, which is not suitable to be represented in an *n*-dimensional linear space. Social networks, for instance, are typically modelled by directed graphs, which are considered to be non-Euclidean examples. The non-Euclidean nature of such data implies that there are no such familiar properties as global parameterisation, common system of coordinates, vector space structure, or shift-invariance. Consequently, basic operations like convolution that are taken for granted in the Euclidean case are even not well defined on non-Euclidean domains [32].

While solutions on Euclidean data are always research topics throughout the history of machine learning, the study of deep learning methods on graph has made some achievements on non-Euclidean structured data in recent years. Thomas N. Kipf et al. proposed *Graph Convolutional Network* (GCN) [33], which is firstly used to train graph data for many tasks. However, GCN considers all 1-hop neighbours of the central nodes equally important, basing on the assumption that connected nodes have similar characteristics. It is unreasonable because it cannot mine the accurate correlation between nodes (in a graph-structured data, a node in graph indicates a sample) and the semantic information under the graph. In order to solve this shortcoming of GCN, Petar Velickovic et al. proposed *Graph Attention Network* (GAT) [34], which attaches different importance to the 1-hop neighbourhood of a central node. We discovered that GAT is capable to distinguish the correlation between nodes well and better extract and propagate features, with the assistance of its attention mechanism (AM). However, GAT only focuses on the 1-hop neighbourhood (that is, the adjacent neighbours) of the central nodes, which drops a big amout of node information in the distance. In the later experiments in our paper, we construct graphs using cancer datasets, and then they are fed into the model for training to obtain cancer prediction. We extended and improved the AMs of GAT by introducing the gating mechanism (GM) similar to *Gated Recursive Unit* (GRU) proposed in [35], and proposed the *Gated Graph ATention network* (GGAT) for cancer prediction. Theoretically, the GGAT is capable to aggregate node information in a multi-hop neighbourhood (that is, the neighbours within multi-step scale) of the central nodes.

One of the benefits of AMs is that they allow variable size input to be processed, focusing on the most relevant part of the input to make decisions. The idea is to calculate the hidden representation of each node in the graph by following a self-focusing strategy and caring for its neighbours [34]. It solves several problems in the previous methods of modeling graphical structure data with neural networks, and has the following advantages:

- **It is highly efficient**. Being aware the fact that the algorithm is based on the two matrices (feature matrix and adjacency matrix) of the graph, the operation of the self-attention may be parallelised across all edges, and the computation of output features can be parallelised across all nodes. There is no need for eigen decomposition or dense matrix calculation.
- **It allows input graphs with different structures**, so that the model can learn automatically without considering the graph structure.
- **It uses node features for similarity calculations**, rather than the structural properties of nodes, in which no need for a prior understanding of the graphical structure.

In our work, we first obtained and processed three cancer datasets LIHC (liver cancer), LUAD (lung adenocarcinoma) and KIRC (renal clear cell carcinoma) from The Cancer Genome Atlas (TCGA) (The Cancer Genome Atlas (TCGA), a landmark cancer genomics program, molecularly characterized over 20,000 primary cancer and matched normal samples spanning 33 cancer types. This joint effort between the National Cancer Institute and the National Human Genome Research Institute began in 2006, bringing together researchers from diverse disciplines and multiple institutions.) for cancer prediction. Then we used our proposed Hybrid Feature Selection algorithm (HFS) to select cancer genes strictly, so as to adapt the characteristics of high latitude and small sample of gene expression profile. Finally, we constructed three graphs using these three datasets, which are fed into the GGAT model proposed by us for training to obtain the cancer prediction. In order to better obtain the correlation between nodes and capture the semantic information under the graph structure, we introduced two kinds of AMs in our GGAT model. One is used to measure the importance between the central nodes and their 1-hop neighbourhood (namely the layer-in AM), which is similar to the AMs proposed by GAT in [34], calculating the similarity between two nodes; the other is used to measure the importance of nodes in the multi-hop neighbourhood of the central nodes (namely the layer-out AM). Here, the GM is introduced to realise the layer-out AM, which retains and forgets the nodes information in the path selectively. Under the combination of the both AMs, our GGAT model is able to aggregate the information of remote nodes, fully exploiting the potential correlation and semantic information among samples. In addition, our GGAT model can be easily applied to all cancer datasets from TCGA and most other graphs. The main contributions of our work are as follows:

1. We propose a hybrid feature selection algorithm to select the gene features, so as to reduce noise and ensure the effectiveness of genes datasets.
2. We propose a novel GGAT for cancer prediction, which can fully mine the semantic information of graph structure (the relevance between samples). According to our knowledge, we are the first to apply GM to graph attentional network.
3. Compared with some of the traditional algorithms and some latest neural network models, our model gains the best accuracy on the experiment datasets. And through the comparative analysis of various experiments, the validity and interpretability of our model are verified.

## 2. Related Work

### 2.1. Machine Learning Methods for Cancer Prediction

Machine learning methods can effectively find relevant patterns in a large number of patient data for cancer analysis and prediction, better considering and combining various factors of cancer data. As is proposed by [36], machine learning methods have been utilised to predict benign ovarian tumors (BOT) and ovarian cancer (OC). Minimum Redundancy Maximum Relevance (MRMR) feature selection method was first used by them to select eight salient features from 235 patients’ data (89 BOT and 146 OC); two of them were identified as the top features by the two-level decision tree: human epididymis protein 4 (HE4) and carcinoembryonic antigen (CEA). Although this is a simple model and only uses two biomarkers, it achieves better classification performance than ovarian malignancy algorithm (ROMA) and logistic regression model. Furthermore, Ref. [37] used machine learning methods to predict cervical cancer. Because of the imbalance of cervical cancer data, it first used three kinds of balancing techniques (ROS, RUS, and SMOTE) to make the data balanced before feeding the data into the model; then it compared the prediction performance of three machine learning methods: k-nearest neighbor, support vector machine, and random forest tree, the results have shown that the random forest tree is the best one. In addition, Ref. [38] constructed a breast cancer prediction learning algorithm based on principal component analysis (PCA) integrating multilayer perceptron network (MLP) and support vector machine (SVM), where PCA is used to reduce the dimension of data and extract the valuable part of data. Then, MLP is used to extract the features contained in the data. In the process of training and learning, the model can separate the representative attributes and numbers automatically, and then use the transfer learning technology of SVM to take the feature data as the classifier.

There are also works showing that the machine learning methods also help doctors provide more accurate prognosis and personalized treatment so as to reduce the cost of each patient. As is proposed by [39], it used Multi-Layer Perceptron (MLP) to predict cancer, with valuable information given as clinical notes included in patient records. It presented an approach to obtain information from clinical notes, based on natural language processing techniques and paragraph vectors algorithms, which is defined as information extraction. By introducing additional information, Ref. [39] obtained better prediction results.

To conclude, the process of traditional machine learning algorithms for cancer prediction is generally divided into two steps: features selection and traditional algorithms selection. Although the features are filtered, these algorithms are generally difficult to mine the semantic information under the graph, to obtain useful information under the connections of logical nodes and to get better prediction results.

### 2.2. Deep Learning Methods for Cancer Prediction

Most machine learning methods cannot fully mine the potential correlation between samples, with less pattern recognition and large amount of calculation. Noticeably, deep learning methods have been developed rapidly, and are also employed in the field of cancer prediction.

Deep learning methods are generally composed of multiple layers. They are widely employed to process large-scaled natural data [20] based on their ability to identify data from different categories. As is described in [40], it proposed a multi-modal neural network integrating Graph Convolutional Networks (GCN) and Deep Neural Network (DNN) to train multi-modal data (including gene expression profile, CNA, DNA methylation and clinical data) for predicting colorectal cancer and its prognosis. In their work, GCN was applied to multi-modal data to predict whether an individual has cancer or not, where the weight of each gene can be scored to select the most effective gene features. These gene features were then fed into DNN separately to intergrate multidimensional data and then to predict the prognosis of human calorectal cancer. It can reduce the dimensions of datasets and consider the heterogeneity of different types of data. The authors of [41] studied the grading of colorectal cancer histology images. It proposed a representative nuclei sampling strategy for transforming an image to a cell graphs can be fed into the GCN, where the nuclei were treated as the nodes and the potential cellular interactions as relations (edges) of the graph. To acquire accurate node features, it applied a nuclear segmentation network and extract appearance features based on the segmented foreground instances. As is shown in [42], GCN-based model has been utilised to combine several heterogeneous omics data types with a graph representation of the data and learn abstract features from both data types, including gene mutation rates, gene DNA methylation, gene expression. The results showed that GCNs are highly suitable to handle multi-dimensional omics data sets and gene-gene interaction networks for cancer prediction. In addition, their model was combined with feature interpretation techniques such as Layer-wise Relevance Propagation method (LRP) to gain meaningful mechanistic insights into how a gene contributes to a specific cancer type. In addition, Ref. [43] also fused of multiple genomic data for cancer prediction, including genetic expression, copy number alteration, DNA methylation and exon expression. It first proposed a similar network fusion algorithm (SNF) to integrate multiple genomic data and clinical data to generate sample similarity matrix. Min-redundancy and max-relevance algorithm (MRMR) were then used to select features to generate sample feature matrix. Finally, the two matrixes were fed into GCN for semi-supervised training to predict cancer. Moreover, Ref. [44] used graph convolutional neural network (GCNN) to predict cancer types and identify cancer specific markers. It proposed four GCNN models for four graphs, namely the co-expression network, the co-expression + singleton network, the PPI network, and the PPI + singleton network. The models proposed successfully classified cancer samples and normal samples, suggesting the markers identified are likely cancer specific without dependency on tissues.

At present, deep learning methods have achieved good results in various fields. However, we discovered that these methods generally only focus on the 1-hop neighbourhood of the central nodes. In addition, many deep learning methods suffer from the problem of gradient vanishing, which means that the gradient of the front layer is smaller than that of the back layer. What is worse, the gradient vanishing problem may cause training to slow down or even stop, and the insufficient training will eventually lead to prediction failure. To fix these flaws, we proposed GGAT model that focus on the multi-hop neighbourhood by introducing the GM similar to GRU [35]. Moreover, by utilising the GM, our model is capable to alleviate the problem of gradient disappearance, by always producing disturbance to allow the training to continue.

## 3. Method

### 3.1. Data Collection and Preprocessing

We used three datasets LIHC (liver cancer), LUAD (lung adenocarcinoma), KIRC (renal clear cell carcinoma) from TCGA and the combination of these three datasets (named Cancer_all) for our experiments.

The values in these datasets are manually collected, which may result in invalid data being recorded. For instance, while the hospital staffs were collecting medical data of cancer patients, some attributes (such as blood type, white blood cell count, red blood cell count, etc.) may be missing due to their omission or negligence, leading to passively supplement the value of 0s in the datasets. Such patient data rows are considered man-made ‘garbage’ which are utterly useless and are completely not descriptive, leading to potential damage of the further experimental results. In fact, these invalid values may have potential, but not always inevitable, influence upon the results of further experiments. To reduce the damage of experimental results to the minimum and ensure the effectiveness and authenticity of our experiments, we employed ‘data cleaning’ techniques to be the process of preparing data for analysis by removing or modifying data rows that are incorrect, incomplete, irrelevant, duplicated, or improperly formatted. Specifically, we processed and normalised the data by deleting the rows with the mean value of 0 in the samples, to make the datasets more suitable to our work.

#### 3.1.1. Feature Selection

To achieve a higher accuracy on cancer prediction tasks, the feature selection procedure is then performed through our Hybrid Feature Selection (HFS) algorithm, which divides the feature selection process into two steps:

(1) **Primary feature selection stage**. In this stage, we filtered irrelevant genes through data difference analysis with fold-change and *p*-value. We utilised edgeR, a software for gene difference analysis, as an assistant. There are two most important steps of edgeR: (1) calculate the difference multiple list (DML); and (2) select the differentially expressed genes by using the edgeR package functions.

The calculated DML includes-

- logFC—Fold Change after log2 transformation. Fold Change, or FC, is the multiple of expression difference between samples;
- logCPM—CPM after log2 transformation. CPM value is used to filter low expression genes;
- LR—likelihood ratio statistics;
- PValue—*p*-value of difference significance; and
- FDR—the *p*-value corrected by FDR.

After the DML calculation process, the differentially expressed genes can be selected according to the calculated DML. The threshold for selection may be modified; in our work, the genes with significant changes are defined as FC (Fold Change) ≥ 2.0 and PValue (*p*-value) < 0.05.

We appoint the genes with significant changes as the genes selected by the primary feature selection stage.

(2) **Exact feature selection stage**. In this stage, we used recursive feature elimination (RFE) [45] algorithm to filter redundant genes by tenfold cross validation. The RFE algorithm is also capable for generating a ranking of the genes, by repeatedly perform the two steps: (1) build the model to select the best (or worst) features according to its configuration; (2) put the selected features aside, and repeat the same process on the remaining features until all the features are traversed. In the end, the order that the features being eliminated in the repeating processes is output as the rank of features. We select the top *N* best features according to the output rankings, where *N* is a parameter being trained during the process of the RFE algorithm.

We performed the aforementioned 2 steps, and there are 14 features from KIRC, 21 features from LIHC, 16 features from LUAD and 75 features from Cancer_all selected by the algorithm. Due to the small number of normal samples, we carried out sample equalization operation by oversampling. The data collection and analysis were performed using the Sangerbox tools, a free online platform for data analysis.

In general, the feature selection algorithms proposed in this section may work as an assistant of any machine learning methods. Experiments in Section 4.3.1 have shown that our feature selection algorithms resulted in significant performance in reducing the training time of algorithms, without noticeably affecting their accuracy.

#### 3.1.2. Graph Construction

In order to build the input graph of the model, we constructed the feature matrix and adjacency matrix of each dataset right after the feature selection procedure. The feature matrix is composed of 3 parts: the sample ID column, selected genes (features) columns, and the label column (suffered from the disease or not). Each row in the feature matrix indicates the experimental fact of an individual participant (a sample) of the dataset.

An adjacency matrix is the connection representation of a graph, which can be indicated by $A\in R^{N\times N}$ where *N* is the number of nodes of a graph. In this work, we constructed the graph where a certain node indicates a certain participant. The value in the *i*th row and the *j*th column of matrix *A*, i.e., $a_{ij}$, equals to 1 if the node (participant) *i* is connected to *j*, or $a_{ij}=0$ if otherwise. In this work, we choose the Euclidean distance (Any method for similarity measurement can be used as measure of connectivity, such as Euclidean Distance, Manhattan Distance, Chebyshev Distance, Minkowski Distance, Mahalanobis Distance, Cosine, Hamming distance, Jaccard similarity coefficient, etc. We chose a method which achieved the best experimental results.) as a measure of the connectivity of nodes—we assume a two nodes is connected if their Euclidean distance value is less than a certain threshold value. The threshold value is a hyperparameter in this work, which we set as the average value of Euclidean distance between all nodes. Moreover, it can also be treated as a tuning parameter in the model for training to obtain a more reasonable and effective threshold through machine learning methods, which is left for our future work.

### 3.2. Gated Graph Attention Network (GGAT)

In Section 3.1.2, we construct graphs to represent the networks of samples (participants) in certain cancer investigations. In order to mine the underlying semantic information of this graph-structured data, we propose GGAT. Similar to what is done in [34], GGAT learns to lie different importance of nodes in every node’s neighbourhood. In addition, we introduce a GM to capture the importance of the remote nodes relative to the central node, along with the AM.

In the Graph Theory, a graph may be denoted by G=(E,A), where *E* represents the set of entities (nodes) and *A* is the adjacency matrix (as defined in Section 3.1.2). Specifically, The input of our model is a set of node features, that is, the feature matrix $e=\left\{{\vec{e}}_{1},{\vec{e}}_{2},\ldots ,{\vec{e}}_{N}\right\},{\vec{e}}_{i}\in R^{F}$ where *N* is the number of nodes, *F* is the number of features of every node and ${\vec{e}}_{i}$ is the feature vector of the entity *i*. The model produces a set of transformed node features vectors $e^{\prime }=\left\{{\vec{e}}_{1}^{\prime },{\vec{e}}_{2}^{\prime },\ldots ,{\vec{e}}_{N}^{\prime }\right\},{\vec{e}}_{i}^{\prime }\in R^{F^{\prime }}$ where ${\vec{e}}_{i}^{\prime }$ is the output embedding of the entity *i*.

#### 3.2.1. 2 Kinds of AMs in GGAT

We introduce 2 kinds of AMs in GGAT in their calculation order:

(1) **Layer-in Attention**. This AM is based on that proposed in [34]. We call it the ‘layer-in attention’, because it learns to lie different importance of nodes in every node’s direct neighbourhood during the aggregation process which happens in every single layer of the model.

As to the layer-in AM of the (l+1)-th GGAT layer (l≥0), the Raw Attention Value of two connected nodes *i* and *j* is defined as:

(1) $$b_{ij}^{\left(l+1\right)}=a\left(W{\vec{h}}_{i}^{\left(l\right)},W{\vec{h}}_{j}^{\left(l\right)}\right)$$

where ${\vec{h}}_{i}^{\left(l\right)}$ and ${\vec{h}}_{j}^{\left(l\right)}$ represent the input embeddings of nodes *i* and *j* respectively. Specifically, the input embedding of each node is initialised by the initial features of the node if the layer is the first layer of the whole model, viz. ${\vec{h}}_{i}^{\left(0\right)}={\vec{e}}_{i}$. $W\in R^{F\times F^{\prime }}$ is a parametrized linear transformation matrix that maps the *F*-dimension input feature vectors to $F^{\prime }$-dimension high-level vectors. a(·,·) is the attentional mapping that maps two $R^{F}$ vectors into a scalar, parametrized by a shared weight vector $\vec{a}\in R^{2F^{\prime }}$. It acts as an ‘attentional enhancer’ in the layer-in attention, that is, it determines the extent to which node *i* aggregates from node *j* in a certain layer of the model.

In order to make comparison on the significance of different nodes, the Raw Attention Value of two connected nodes *i* and *j* ($b_{ij}^{\left(l+1\right)}$) is then are normalised by Equation (2), to obtain the Normalised Attention Value ($\alpha_{ij}$):

(2) $$\alpha_{ij}^{\left(l+1\right)}={\mathrm{softmax}}_{j}\left(b_{ij}^{\left(l+1\right)}\right)=\frac{\exp\left(b_{ij}^{\left(l+1\right)}\right)}{\sum_{k\in N_{i}}\exp\left(b_{ik}^{\left(l+1\right)}\right)}$$

where $N_{i}$ represents the set of node *i*’s direct neighbours, or nodes that have connection with node *i* (including node *i* itself).

Then, we make use of the Normalised Attention Values ($\alpha_{ij}^{\left(l+1\right)}$s) to calculate the linear combination of their corresponding neighbours’ features as the final output features of each node. Specifically, for a certain node *i*, its Attention-ed Embedding is calculated by Equation (3):

(3) $${\vec{m}}_{i}^{\left(l+1\right)}=\sigma \left(\sum_{j\in N_{i}}\alpha_{ij}^{\left(l+1\right)}W{\vec{h}}_{j}^{\left(l\right)}\right)$$

where σ(·) is a non-linearity function mapping each dimension of the aggregated node representations to be in the range (0,1); we use softmax as σ(·) in our work. $W\in R^{F\times F^{\prime }}$ is a shared weight matrix in linear transformation.

Additionally, we employ multi-head attention for stabling the learning process of self-attention, similarly to [34,46]. Specifically, the transforms in Equations (1)–(3) are calculated *K* times independently, and the resulting vectors are concatenated eventually. Finally, the Multi-Head Attention-ed Embedding of a certain node *i* is described as:

(4) $${\vec{m}}_{i}^{\left(l+1\right)}={∥}_{k=1}^{K}\sigma \left(\sum_{j\in N_{i}}\alpha_{ij}^{k,(l+1)}W^{k}{\vec{h}}_{i}^{\left(l\right)}\right)$$

where ∥ represents the concatenation operation of vectors, $\alpha_{ij}^{k,(l+1)}$ is the Normalized Attention Value of node *i* to *j* calculated by the *k*-th AM [Equation (2)], $W^{k}$ is the corresponding AM’s weight matrix of the input linear transformation. Being aware that concatenation is no longer sensible in the output part, as described in [34], averaging is employed to calculate the Multi-Head Attention-ed Embedding if the multi-head attention layer is the endmost attention layer of a GGAT model [Equation (5)].

(5) $${\vec{m}}_{i}^{\left(l+1\right)}=\sigma \left(\frac{1}{K}\sum_{k=1}^{K}\sum_{j\in N_{i}}\alpha_{ij}^{k,(l+1)}W^{k}{\vec{h}}_{i}^{\left(l\right)}\right)$$

Despite the fact that the aforementioned techniques could successfully aggregate information from adjacent neighbours selectively, we discovered that the layer-in attention drops all structural information, and merely considers the 1-hop neighbourhood (i.e., adjacent neighbours) of each node, which means the information that sealed in remote nodes is ignored. In an attempt to allow the nodes to aggregate information from wider scopes, we extend the model with the subsequent layer-out attention technique.

(2) **Layer-out Attention**. We introduce a GM, similar to *Gated Recurrent Unit* (GRU) proposed in [35], to capture and filter the importance of the remote nodes related to the central node. This method allows the model to capture information from *remote* nodes selectively, which plays an important part between layers. We named it the ‘layer-out attention’ based on this distinguishing characteristic.

In semantic mining tasks of graph-structured data, for any node in the graph, the information from remote neighbours is always important—they make an indispensible contribution in classifying the central node. If the importance of these remote nodes can be taken into account at the same time, the semantic information of the *whole* graph structure may be digged out. Thus, for the purpose of extending the ‘field of vision’ of nodes in layer-in attention, we try stacking multiple layer-in attention layers, and introduce our layer-out attention layers in between.

In layer-out AM, information coming from remote nodes may either pass through or be blocked by the gates. The gates of the layer-out AM, in particular, consists of three individual gates, viz. the Update Gate, Reset Gate and Forget Gate. Initially, the input embedding (${\vec{h}}_{i}^{\left(l\right)}$) and the (Multi-Head) Attention-ed Embedding (${\vec{m}}_{i}^{\left(l+1\right)}$) of each specified node are fed into the Update Gate and Reset Gate simultaneously, to calculate two Gated Node Representations:

(6) $${\vec{z}}_{i}^{\left(l+1\right)}=\sigma \left(W^{z}{\vec{m}}_{i}^{\left(l+1\right)}+U^{z}{\vec{h}}_{i}^{\left(l\right)}\right)$$

(7) $${\vec{r}}_{i}^{\left(l+1\right)}=\sigma \left(W^{r}{\vec{m}}_{i}^{\left(l+1\right)}+U^{r}{\vec{h}}_{i}^{\left(l\right)}\right)$$

where ${\vec{z}}_{i}^{\left(l+1\right)}$ and ${\vec{r}}_{i}^{\left(l+1\right)}$ are node *i*’s Gated Node Representations of the Update Gate and the Reset Gate respectively, $W^{z}$, $W^{r}$, $U^{z}$ and $U^{r}$ are weight parameter matrices to be trained. The update gate is aimed at controlling information forgetting, and the reset gate is employed in controlling information updating.

The Gated Node Representations comming out from the Reset Gate of node *i* are then applied to Equation (8) to obtain a New Information Vector:

(8) $${\vec{n}}_{i}^{\left(l+1\right)}=\tanh\left(W_{n}{\vec{m}}_{i}^{\left(l+1\right)}+U_{n}\left({\vec{r}}_{i}^{\left(l+1\right)}⊙{\vec{h}}_{i}^{\left(l\right)}\right)\right)$$

where the operator ⊙ denotes the Hadamard Product of two vectors of the same dimension, and $W_{n}$ and $U_{n}$ are weight parameter matrices to be trained.

Finally, the input embedding, along with the New Information Vector of node *i*, are fed into the Forget Gate (Equation (9)) to obtain the Gated Embedding of the node

(9) $${\vec{h}}_{i}^{\left(l+1\right)}=\left(1-{\vec{z}}_{i}^{\left(l+1\right)}\right)⊙{\vec{h}}_{i}^{\left(l\right)}+{\vec{z}}_{i}^{\left(l+1\right)}⊙{\vec{n}}_{i}^{\left(l+1\right)}$$

where $\left(1-{\vec{z}}_{i}^{\left(l+1\right)}\right)⊙$ and ${\vec{z}}_{i}^{\left(l+1\right)}⊙$ are working as selectors to choose information to be forgotten and remembered respectively. ${\vec{h}}_{i}^{\left(l+1\right)}$ is the Gated Output Embedding of node *i*, which is the output feature vector of the (l+1)-th GGAT layer with respect to node *i*.

#### 3.2.2. Demonstration of the GGAT’s Operation

**Figure 1:**
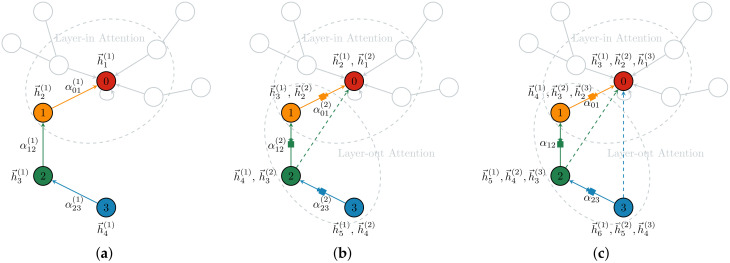
The GGAT Architecture (Shown in Single Head Attention). (**a**–**c**) are ordered according to the layer number, i.e., (**a**–**c**) demostrate the information holding status within the first, second and third layer of the Gated Graph ATtention network (GGAT) model, respectively. The labels nearing the coloured nodes indicate the sources of their accumulated information in the current step. For instance, by the end of the second layer’s accumulation process (**b**), the green node possesses information from ${\vec{h}}_{4}^{\left(1\right)}$ and ${\vec{h}}_{3}^{\left(2\right)}$ which was gathered from the blue node in the second (current) layer. Likewise, as is shown in (**c**), the red node owns information from ${\vec{h}}_{3}^{\left(1\right)}$, ${\vec{h}}_{2}^{\left(2\right)}$ and ${\vec{h}}_{1}^{\left(3\right)}$ which was gathered from the orange node in the third layer.

By stacking multiple layer-in AM and layer-out AM, the whole GGAT model is capable of capturing multi-hop (remote) information of any nodes. We discovered that our GGAT model achieved the best performance when stacking three GGAT layers, that is, an initial GGAT layer without layer-out attention (gates), and two GGAT layers with layer-out attention (gates). According to our model structure, we consider the information aggregation process in a 3-hop neighbourhood throughout this study as is shown in Figure 1.

In the initial layer (Figure 1a), only the layer-in attention is calculated because there is no gates. It calculates the summation of the importance of all 1-hop neighbours (adjacent nodes) of the central nodes. The input of the first layer is the feature vectors (*d*-dimension) of the nodes (i.e., the columns of the feature matrix). After the forwarding propagation of the initial GGAT layer, the output feature vector (*k*-dimension) of the node (${\vec{h}}_{i}^{\left(1\right)}$), calculated by the layer-in AM, is obtained. This representation is then fed into the log-softmax classifier to generate the classification vector (*k*-dimension), which is the output vector of the first layer.

In the second layer (Figure 1b), the layer-in attention and the layer-out attention are all calculated. The input of the second layer is ${\vec{h}}_{i}^{\left(1\right)}$ (*k*-dimension) from the first layer. After the forwarding propatation of the second GGAT layer, a new output feature vector of the node (*k*-dimension), calculated by both the layer-in AM and the layer-out AM (shaped like a filter in Figure 1), is obtained. The output feature vector (*k*-dimension) is then be fed into the final log-softmax of this GGAT layer to generate the output vector (*k*-dimension) of this layer, which is the output vector of the second layer, or the input of the third layer.

The operations in the third layer (Figure 1c) is the identical as the aforementioned second layer. Here we take the knowledge graph in Figure 1 as an example to explain our model.

The node, coloured red and marked **0**, is the central node. For clarity of presentation, a special path that passes through a 1-hop neighbour, a 2-hop neighbour and a 3-heighbour of the central node is considered, i.e., the depicted path that passes through the nodes marked **0**, **1**, **2** and **3**. The orange node, marked **1**, is the 1-hop neighbour of the red node. The green node, marked **2**, is the 2-hop neighbour of the red node. The blue node, marked **3**, is the 3-hop neighbour of the red node.

As an extension to the Figure 1, an example of information contained in each node in the three-layer model propagation is shown in Table 1.

- In the first layer, each node aggregates its own and 1-hop neighbours’ information using layer-in attention. At this point, the Node 0 aggregates its own information 0 and its neighbour Node 1’s information 1, so it contains information 0, 1; the Node 1 aggregates its own information 1 and its 1-hop neighbour Node 2’s information 2, so it contains information 1, 2; Node 2 and Node 3 are similar to Node 1.
- In the second layer, each node aggregates via layer-in attention and layer-out attention simultaneously. At this point, in addition to the information 0, 1 collected in the first layer, Node 0 continues to aggregate the information of its neighbour Node 1 filtered by the gates, i.e., (1, 2) (The parentness indicates a transform by the gates. For example, (1, 2) indicates the gated version of information from 1, 2; ((3, 4)) indicates the information from 3, 4 that is filtered by the gates twice), so Node 0 contains information 0, 1, (1, 2) at this time. In addition to the information collected in the first layer 1, 2, Node 1 continues to aggregate the information of its neighbour Node 2 filtered by the gates, that is, (2, 3), so Node 1 contains information 1, 2, (2, 3) currently; Node 2 and Node 3 are similar to Node 1.
- The operation of the third layer is the identical to that of the second layer.

It can be seen that in the second layer, the information from the second-order domain (i.e., the 2-hop neighbourhood) can be aggregated by any nodes. In the information aggregation process of the third layer, likewise, each node is capable of aggregating information from its third-order domain.

#### 3.2.3. Other Facts about GGAT

The GGAT is equipped with the back propagation technique to do parameter fine-tuning, just as other machine learning methods do. The loss of classification result of each node in the network is calculated via nll-loss (Negative Logistic Loss) function, and the gradients are calculated based on the summation of the losses. As a result, the parameters are fine-tunned with respect to the calculated gradients, in a gradient-descent manner. The fine-tunning operations are done by the Adam optimiser automatically in each training epoch of the GGAT.

To conclude, the GGAT model is capable of aggregating the important information in the multi-hop neighbourhood from the central nodes selectively, thus the correlation between nodes may be considered to the full. To conclude, by introducing the AMs and GMs, our model obtains the ability to:

1. measure the dependence of long-distance nodes;
2. take into account the different importance of each node in multi-hop neighbourhood;
3. aggregate as much semantic information as possible; and
4. fully mine the correlation between samples.

We argue that all these abilities can intuitively improve the cancer prediction accuracy with the assistance from deep learning techniques.

## 4. Experiment

### 4.1. Experiment Design and Setup

All experiments are performed in a unified server environment (We performed all experiments on a Dell PowerEdge T640 workstation, running Ubuntu 16.04, with 2 Intel Xeon Silver 4210 1 GHz CPUs and 2 Nvidia GeForce GTX 1080 Ti GPUs; all codes are implemented by the PyTorch framework). As for model configuration, the overall learning rate was set to 0.005, the number of hidden units was set to 8, and the number of attention heads was set to 8. We discovered that these configurations give the best experimental results.

Several groups of experiments were carried out as follows.

- **Evaluation on the Hybrid Feature Selection Algorithm**. We performed the prediction task over different versions of feature numbers, of KIRC, LIHC, LUAD [including the Optimal Feature Number (OFN), 50, 100, 200, 500, 1000, 2000, and Initial Feature Number(IFN) versions] and Cancer_all (including OFN and IFN versions) to verify the effectiveness of our proposed hybrid feature selection algorithm (Section 3.1.1).
- **Evaluation on the GGAT’s Performance**. We compared the accuracy (%) and training time (seconds) among GGAT and other models. In order to verify the effectiveness and performance of our GGAT model, we also utilised the combined cancer dataset (Cancer_all) to investigate the accuracy and training time of the multi-category classification task (3 categories) under large data scale.
- **Evaluation on the GGAT’s Validity and Interpretability**. Among these models for comparison, the traditional machine learning methods include K-Nearest Neighbor, Support Vector Machine and Decision Tree, and neural network models include GCN and GAT.

All experiments were carried out ten times and the final results were averaged.

### 4.2. Introduction of Datasets

The cancer prediction task in our work is considered a node classification task, because we built cancer datasets into a graph to predict whether a node (a sample of participant) has cancer or not. We used LIHC (liver cancer), LUAD (lung adenocarcinoma), KIRC (renal clear cell carcinoma) datasets from TCGA and their combination dataset Cancer_all for our experiments.

For KIRC, LIHC and LUAD datasets, they are randomly shuffled before being split into training, validation and testing sets. After the shuffle, each of them is then divided into the three sub-sets, proportionally—the top 40% entries of the shuffled dataset become training sets, the latter 20% entries become testing sets, and the rest 20% are appointed as validation sets. We believe that through shuffle, the proportion of cancer samples and normal samples are approximately the identical in the three sets.

The feature dimension information of these datasets before and after our hybrid feature selection algorithm is shown in Table 2. It is clear that for Cancer_all dataset, there are no normal samples—in fact, this combined dataset is used to do multi-class classification as to catalogue all samples into three cancer catagories (KIRC, LIHC and LUAD). The division of this dataset is likewise—the top 40% entries of the shuffled dataset become training sets, the latter 20% entries become testing sets, and the rest are appointed as validation sets.

In addition, being aware the fact that Cora is a widely used benchmark dataset for node classification task, we to perform a supplementary experiment on Cora to verify the effectiveness of our proposed model GGAT.

All dataset used in our experiments and their details are shown in Table 3, and the information of the constructed graphs from these datasets are shown in Table 4 (The source of the three datasets (KIRC, LUAD and LIHC) are separated; each dataset is the resulting data from a single research. The possibility of the situation where samples from one participant be in multiple datasets is extremely low).

### 4.3. Experimental Results

#### 4.3.1. Effectiveness of the Hybrid Feature Selection Algorithm

**Figure 2:**
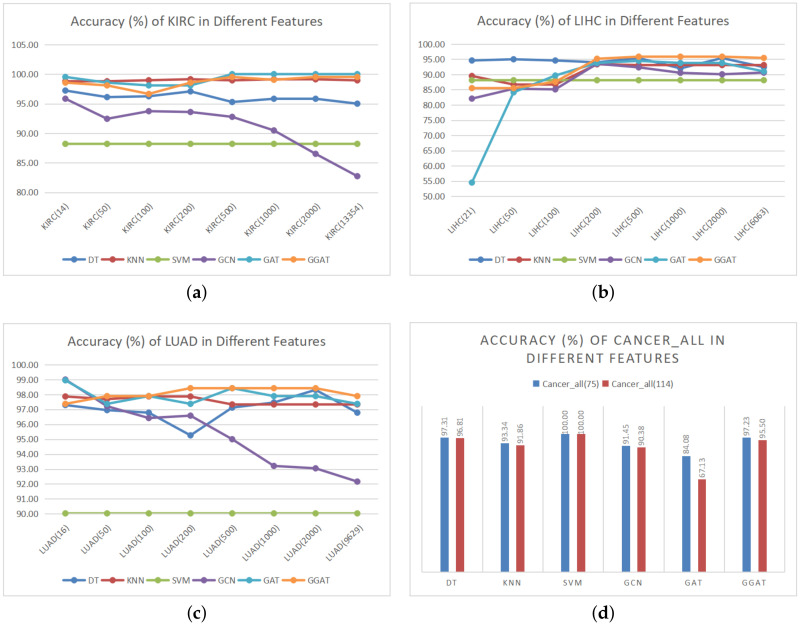
Accuracy (%) of Datasets in Different Features. The digit after the dataset name represents the number of features, the first digit is the optimal feature number selected by our hybrid feature selection algorithm, the last digit is the original feature number before exact selection.

**Figure 3:**
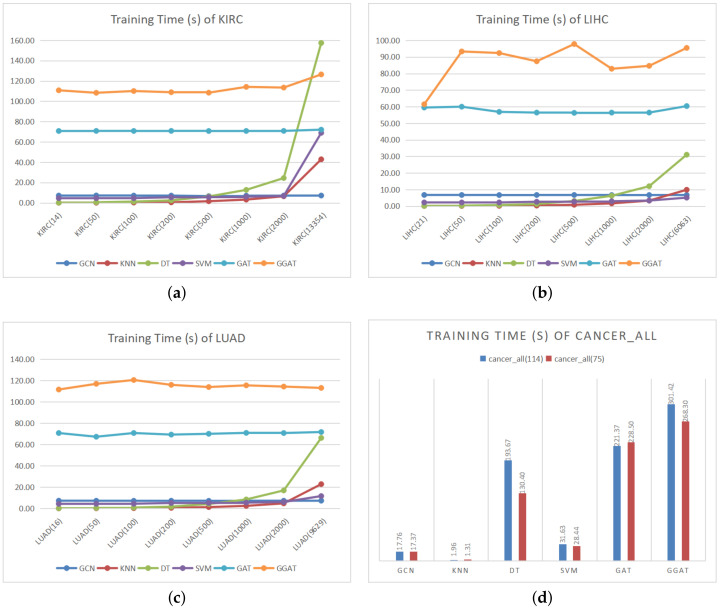
Training Time (s) of Datasets.

In order to verify the effectiveness of proposed hybrid feature selection algorithm, experiments were carried out on datasets with different feature dimensions. The experimental results are shown in Figure 2 (accuracy (%)) and Figure 3 (training time (seconds)).

(1) **Accuracy Comparison**

As is shown in Figure 2, the datasets after feature selection have achieved better accuracy in most models’s training results, which is considered the direct proof of the effectiveness of our hybrid feature selection algorithm. In addition, we discovered that

1. Our hybrid feature selection algorithm has no effect on SVM. We suppose that the way we construct the graph makes the datasets unfriendly to SVM. It has been pointed out in preceding section that we chose the average Euclidean distance to measure whether there is a link between two nodes, whereas the graph constructed by the average value may not be suitable for every model. In the future research, this threshold can be used as a parameter to train and optimize, so as to achieve the value of the specific model, which makes our feature selection algorithm more universal.
2. GCN does not clearly show the advantage of our feature selection algorithm in LIHC. Firstly, we found that the LIHC dataset resulted in more unstable experimental accuracy in multiple models, compared to other datasets: the error rate of which is relatively high (within the acceptable range). Furthermore, there may be the same problems as described in the above point.
3. GAT and GGAT do not highlight the advantages of our feature selection algorithm as well. We hold the belief that feature selection is for better information extraction. However, as a matter of fact, both GAT and GGAT utilise the AM, which is equivalent to feature selection in an indirect manner, that is, the AM in the both models plays the same role as feature selection. In addition, our model introduces GM, which may help to carry out better attention through rigorous information selection and filtering.

(2) **Training Time Comparison**

As is shown in Figure 3, our feature selection algorithm is capable to reduce the training time of models, especially for the traditional machine learning algorithms. It can be seen that the feature selection of the datasets in the early stage greatly improved the performances of the general machine learning algorithms. For the neural network models, there is no significant difference in training time under different feature dimensions—we suppose it is the neural network models’ weight matrices that play significant rows as feature selection when performing linear transforms. In addition, given the introduction of gates, it can be seen that the training time of GGAT was relatively high. However, we argue that it still achieved the best efficiency in the acceptable training time range.

Generally speaking, notwithstanding that our feature selection algorithms did not demonstrably improve the efficiency of all models, they could shorten the training time without reducing the performance of the models.

#### 4.3.2. Effectiveness of GGAT

We compare the performances of each model on datasets mentioned above with different feature dimensions. The results are as shown in Table 5, Table 6, Table 7, Table 8, Table 9, Table 10, Table 11, Table 12, Table 13 and Table 14, where the best result values are shown in bold. Specifically, for sensitivity and specificity comparisons (Table 6 and Table 8), the result values with the highest summation of the both values among those listed models are defined as the best ones.

By analysing the results in Table 5, Table 6, Table 7, Table 8, Table 9, Table 10, Table 11, Table 12, Table 13 and Table 14, we draw the following conclusions:

1. In general, to the best of our knowledge, our proposed model GGAT achieves the best overall results on most of the datasets, which verifies the effectiveness of our model. Specifically, the comparisons on sensitivity and specificity show that our model achieves the stablest effects on predicting three cancer types, among the listed graph convolution models.
2. The previous data processing method and the characteristics of the datasets itself indeed improved the effectiveness of a number of certain algorithms, e.g., SVM on Cancer_all and GAT on KIRC.
3. The more features, the higher effectiveness we found of GAT and GGAT. As is mentioned above, both the GAT and GGAT introduce AM which play the same role as feature selection. Therefore, more information may be selected and aggregated by the AM if more features are given, thus better results may be obtained. In particular, our model introduces gates, which assists to select and filter information that is more important and effective, and to make the node classification more accurate.
4. On the contrary, the more features, the lower efficiency of the traditional algorithm. A conclusion can be drawn that the feature selection algorithm may greatly help the traditional classification algorithms to learn and calculate more effectively, and most importantly, reduce the training time.

To conclude, our aim is to provide a simple and accurate approach to reduce the doctors’ works. It shall be pointed out that our method provides helps to doctors when they are making diagnosis, instead of making diagnosis for doctors. The experiments in this section shows that the prediction accuracy is sufficiently high, despite the fact that no research on pathology and histology is performed.

#### 4.3.3. Evaluation on the Interpretability of GGAT

We studied the experiments and further investigated the validity and interpretability of our proposed GGAT model. We point out 2 major claims on the basis of the validity and interpretability study.

(1) **The gates can help solve the problem of gradient disappearance**.

**Figure 4:**
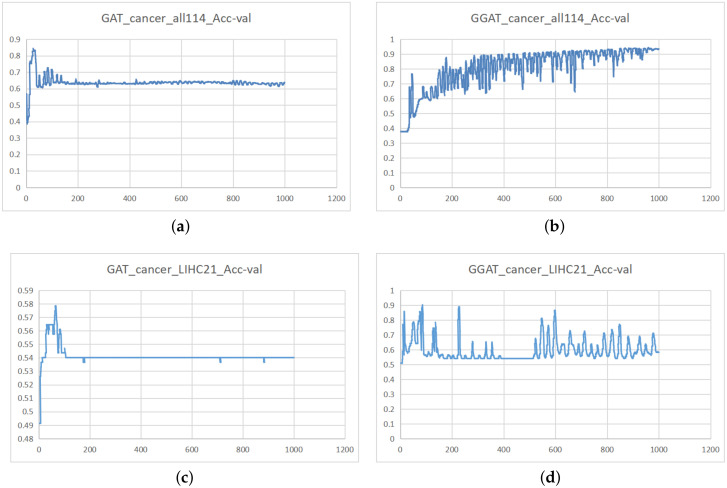
Acc_val of GAT (**left**) and GGAT (**right**) When Training on Cancer_all and LIHC(21).

In the process of experiments, we noticed that the accuracy of GAT was extremely low in Cancer_all and LIHC(21), while the GGAT displayed a normal performance (as depicted in Figure 4a,b). By analysing the Acc_val (Accuracy of validation Set) of both GAT and GGAT in training on Cancer_all. It can be seen that the accuracy value of the GAT on Cancer_all dataset almost stop changing after 200 epochs (Figure 4a), whereas the same accuracy value of GGAT continued fluctuating in the inspection epoch range (Figure 4b). This phenomenon indicates that our proposed GGAT is capable to break through the bottleneck of a training process.

According to the claims in [35], the reset gate in GRU is capable of helping their model get rid of gradient disappearing problems. We believe that in our model, it is the reset gate that always produces tiny disturbance during training, resulting in chances to make the training process to continue.

(2) **The AMs of GAT and GGAT can act as feature selection**

As mentioned in Section 4.3.1, GAT and GGAT achieved better experimental results without feature selection. We own this success to the introduction of AM, which plays the same role as feature selection. The AM distinguishes the importance of all nodes to effectively extract and aggregating information.

## 5. Conclusions and Future Work

In this work, we proposed a novel three-layer GGAT, and used it for cancer prediction tasks. Tradition methods shows that certain methods are suitable for certain types of cancer. However, our proposed GGAT model aims at doing the predicting things better—we tried to increase the accuracy on all types of cancer, moderating the difference of the predicting accuracy among different cancer types. From what can be seen in the experiments, the GGAT is capable of aggregating the information in the 3-hop neighbourhood of every node with different importance. By introducing the GM, the information coming from remote nodes may be selectively filtered. In this way, GGAT can comprehensively mine the semantic information under the graph structure and the association between nodes, and provide a more powerful guarantee for cancer prediction. Through the experimental analysis and comparison on various datasets, GGAT had improved the performance by 1–2%, in the acceptable training time range. Moreover, the validity and interpretability of GGAT were verified. In addition, we also proposed and verified the effectiveness of our hybrid feature selection algorithm. Experiments show that our feature selection algorithms resulted in significant performance in reducing the training time of certain algorithms, without noticeably affecting their accuracy.

There are several potential improvements and extensions to our work that could be addressed as future work. We noticed that our ideas of improving the neural network model are based on the graph structure itself, and the semantic information of nodes themselves and additional information outside the graph are temporarily ignored. In the future research, we will focus on the prior knowledge of graph and node attributes, so as to increase the difference between nodes and make the task of nodes on the graph more accurate. In addition, due to the serious imbalance of the positive and negative samples in our datasets, we will also study on the ways to balance data. We are planning to utilise the Generative Adversarial Networks (GAN) and/or other generative models to generate adversarial samples.

## Figures and Tables

**Table 1:** An Example: Information for each node in per layer.

Layer/Node	0	1	2	3
**First Layer**	0, 1	1, 2	2, 3	3, ♡
**Second Layer**	0, 1, (1, 2)	1, 2, (2, 3)	2, 3, (3)	3, ♡, (♡)
**Third Layer**	0, 1, (1, 2),(1, 2, (2, 3))	1, 2, (2, 3),(2, 3, (3))	2, 3, (3), (3, ♡, (♡))	3, ♡, (♡)

**Notes:** The digits in the header indicate the Node Number; the values in each row represent the information within nodes when the corresponding layer outputs. For instance, the Node 0 contains information 0, 1, (1, 2) in the second layer, i.e., the output vector of 0 in the second layer consists of the un-gated information from Node 0 and 1, and 1-time-gated information from Node 1 and 2; the parentness indicates a transform by the gates. For example, (1, 2) indicates the gated version of information from 1, 2, and ((3, 4)) indicates the information from 3, 4 that is filtered by the gates twice; ♡ represents the information of the nodes not listed.

**Table 2:** Feature Dimension of Datasets.

Dataset	After Preprocessing	After Primary Selection (Original Feature Number)	After Exact Selection (Optimal Feature Number)
**KIRC**	610 × 35,208	610 × 13,354	610 × 14
**LIHC**	421 × 29,609	421 × 6063	421 × 21
**LUAD**	592 × 35,734	592 × 9629	592 × 16
**Cancer_all**	1442 × 114	1442 × 114	1442 × 75

**Notes:** The shape of each dataset is row × col, where row is the number of examples and col is the feature dimension. KIRC, LIHC and LUAD are used to cancer prediction (two-classifications). Cancer_all is the dataset combined with KIRC, LIHC and LUAD and left 114 features for cancer prediction (three-classifications).

**Table 3:** Datasets Information.

Dataset	Shape	Cancer Samples	Normal Samples	Classes
**KIRC**	610 × 500	538	72	2
**LIHC**	421 × 500	371	50	2
**LUAD**	592 × 500	533	59	2
**Cancer_all**	1442 × 75	1442	0	3
**Cora**	2708 × 1433	0	2708	7

**Notes:** We take 500 feature dimensions as an example for KIRC, LIHC and LUAD, and 75 feature dimensions for cancer_all.

**Table 4:** Graph Information.

Dataset	Nodes	Edges	Training Set	Validation Set	Test Set
**KIRC**	610	105,428	40%	40%	20%
**LIHC**	421	55,155	40%	40%	20%
**LUAD**	592	107,975	40%	40%	20%
**Cancer_all**	1442	638,719	40%	40%	20%
**Cora**	2708	5429	140 entities	300 entities	1000 entities

**Table 5:** Accuracy of Models Under Optimal Feature Dimensions.

Model/Datasets	KIRC(14)	LIHC(21)	LUAD(16)	Cancer_all(75)	Cora
**DT**	97.22	**94.60**	97.29	97.31	-
**KNN**	98.79	89.51	97.86	91.86	-
**SVM**	88.20	88.12	90.03	**99.00**	-
**GCN**	95.84	82.07	**98.99**	91.45	83.40
**GAT**	**99.52**	54.48	98.95	84.08	84.30
**GGAT**	98.56	85.52	97.37	97.23	**85.40**

**Table 6:** Sensitivity vs. Specificity (TPR vs. TNR, %) of three Graph Convolution Models Under Optimal Feature Dimensions.

Model/Datasets	KIRC(14)	LIHC(21)	LUAD(16)
**GCN**	97.05/94.23	83.32/86.70	98.79/98.54
**GAT**	99.07/100.0	15.38/100.0	**98.23/100.0**
**GGAT**	**100.0/99.06**	**73.08/100.0**	100.0/96.46

**Table 7:** Accuracy (%) of Models Under Original Feature Dimensions.

Model/Datasets	KIRC(13354)	LIHC(6063)	LUAD(9629)	Cancer_all(114)
**DT**	95.00	92.40	96.78	96.81
**KNN**	98.95	93.09	97.33	93.34
**SVM**	88.20	88.12	90.03	**99.00**
**GCN**	82.73	90.62	92.16	90.38
**GAT**	**100.00**	91.03	97.37	67.13
**GGAT**	**99.52**	**95.44**	**97.89**	95.50

**Table 8:** Sensitivity vs. Specificity (TPR vs. TNR, %) of three Graph Convolution Models Under Original Feature Dimensions.

Model/Datasets	KIRC(13354)	LIHC(6063)	LUAD(9629)
**GCN**	92.02/74.26	91.00/90.28	82.72/90.14
**GAT**	**100.0/100.0**	86.57/94.87	98.70/96.46
**GGAT**	99.06/100.0	**98.51/93.59**	**98.70/97.35**

**Table 9:** Accuracy (%) of Models Under 50 Feature Dimensions.

Model/Datasets	KIRC(50)	LIHC(50)	LUAD(50)
**DT**	96.11	**95.00**	96.95
**KNN**	**98.79**	86.70	97.69
**SVM**	88.20	88.12	90.03
**GCN**	92.44	85.31	97.21
**GAT**	98.56	84.14	97.37
**GGAT**	98.09	85.52	**97.89**

**Table 10:** Accuracy (%) of Models Under 100 Feature Dimensions.

Model/Datasets	KIRC(100)	LIHC(100)	LUAD(100)
**DT**	96.25	**94.60**	96.78
**KNN**	**98.97**	89.70	97.86
**SVM**	88.20	88.12	90.03
**GCN**	93.73	85.11	96.42
**GAT**	98.09	89.66	97.89
**GGAT**	96.65	87.59	**97.89**

**Table 11:** Accuracy (%) of Models Under 200 Feature Dimensions.

Model/Datasets	KIRC(200)	LIHC(200)	LUAD(200)
**DT**	97.08	94.00	95.26
**KNN**	**99.14**	93.35	97.86
**SVM**	88.20	88.12	90.03
**GCN**	93.59	93.52	96.58
**GAT**	98.09	93.79	97.37
**GGAT**	98.56	**95.17**	**98.42**

**Table 12:** Accuracy (%) of Models Under 500 Feature Dimensions.

Model/Datasets	KIRC(500)	LIHC(500)	LUAD(500)
**DT**	95.28	95.40	97.12
**KNN**	98.95	93.10	97.33
**SVM**	88.20	88.12	90.03
**GCN**	92.77	92.34	95.00
**GAT**	**100.00**	94.48	98.42
**GGAT**	99.52	**95.86**	**98.42**

**Table 13:** Accuracy (%) of Models Under 1000 Feature Dimensions.

Model/Datasets	KIRC(1000)	LIHC(1000)	LUAD(1000)
**DT**	95.83	92.00	97.46
**KNN**	99.12	93.10	97.33
**SVM**	88.20	88.12	90.03
**GCN**	90.48	90.55	93.21
**GAT**	**100.00**	93.79	97.89
**GGAT**	99.04	**95.86**	**98.42**

**Table 14:** Accuracy (%) of Models Under 2000 Feature Dimensions.

Model/Datasets	KIRC(2000)	LIHC(2000)	LUAD(2000)
**DT**	95.83	95.40	98.31
**KNN**	99.12	93.10	97.33
**SVM**	88.20	88.12	90.03
**GCN**	86.51	90.07	93.05
**GAT**	**100.00**	93.79	97.89
**GGAT**	99.52	**95.86**	**98.42**

## Data Availability

The code implementation and preprocessed datasets of this study are accessible on GitHub (https://github.com/lhanlhanlhan/ggat (accessed on 28 December 2020)). The TCGA datasets were downloaded and cleansed by SangerBox (http://www.sangerbox.com/tool (accessed on 28 December 2020)).

## References

[1] Bray F., Ferlay J., Soerjomataram I., Siegel R.L., Torre L.A., Jemal A. (2018). Global cancer statistics 2018: GLOBOCAN estimates of incidence and mortality worldwide for 36 cancers in 185 countries. CA Cancer J. Clin..

[2] Ning W., Shuo L., Lei Y., Xi Z., Yan-nan Y., Hui-chao L., Jia-fu J. (2019). Interpretation on the report of Global Cancer Statistics 2018. Electron. J. Compr. Cancer Ther..

[3] Rongtao Z., Changli Z., Zhangxian Y. (2008). Ten Methods of Traditional Chinese Medical Cancer Prevention. Mod. Distance Educ. Chin. Tradit. Chin. Med..

[4] Yifu G. (2014). Review of the application and advantages and disadvantages of sequencing technology in gene diagnosis. Hereditas:bjing.

[5] Demsar J., Zupan B., Kattan M.W., Beck J.R., Bratko I. (1999). Naive Bayesian-based nomogram for prediction of prostate cancer recurrence. Stud. Health Technol. Inform..

[6] Hong J.H., Cho S.B. (2006). Multi-class cancer classification with OVR-support vector machines selected by naive Bayes classifier. Proceedings of the International Conference on Neural Information Processing.

[7] Sarkar M., Leong T.Y. Application of K-nearest neighbors algorithm on breast cancer diagnosis problem. Proceedings of the AMIA Symposium. American Medical Informatics Association.

[8] Yoo S.H., Cho S.B. (2004). Optimal gene selection for cancer classification with partial correlation and k-nearest neighbor classifier. Proceedings of the Pacific Rim International Conference on Artificial Intelligence.

[9] Li C., Zhang S., Zhang H., Pang L., Lam K., Hui C., Zhang S. (2012). Using the K-nearest neighbor algorithm for the classification of lymph node metastasis in gastric cancer. Comput. Math. Methods Med..

[10] Jerez-Aragonés J.M., Gómez-Ruiz J.A., Ramos-Jiménez G., Muñoz-Pérez J., Alba-Conejo E. (2003). A combined neural network and decision trees model for prognosis of breast cancer relapse. Artif. Intell. Med..

[11] Razavi A.R., Gill H., Åhlfeldt H., Shahsavar N. (2007). Predicting metastasis in breast cancer: Comparing a decision tree with domain experts. J. Med. Syst..

[12] Yeh J.Y., Wu T.H. (2010). Cascade of genetic algorithm and decision tree for cancer classification on gene expression data. Expert Syst..

[13] Lee Y.J., Mangasarian O., Wolberg W. (2000). Breast cancer survival and chemotherapy: A support vector machine analysis. DIMACS Ser. Discret. Math. Theor. Comput. Sci..

[14] Liu W., Shen P., Qu Y., Xia D. Fast algorithm of support vector machines in lung cancer diagnosis. Proceedings of the International Workshop on Medical Imaging and Augmented Reality.

[15] Liu H., Zhang R., Luan F., Yao X., Liu M., Hu Z., Fan B.T. (2003). Diagnosing breast cancer based on support vector machines. J. Chem. Inf. Comput. Sci..

[16] Valentini G., Muselli M., Ruffino F. (2004). Cancer recognition with bagged ensembles of support vector machines. Neurocomputing.

[17] Nguyen H.N., Vu T.N., Ohn S.Y., Park Y.M., Han M.Y., Kim C.W. (2006). Feature elimination approach based on random forest for cancer diagnosis. Proceedings of the Mexican International Conference on Artificial Intelligence.

[18] Okun O., Priisalu H. (2007). Random forest for gene expression based cancer classification: Overlooked issues. Proceedings of the Iberian Conference on Pattern Recognition and Image Analysis.

[19] Maliha S.K., Ema R.R., Ghosh S.K., Ahmed H., Mollick M.R.J., Islam T. (2019). Cancer Disease Prediction Using Naive Bayes, K-Nearest Neighbor and J48 algorithm. Proceedings of the 2019 10th International Conference on Computing, Communication and Networking Technologies (ICCCNT).

[20] Toğaçar M., Ergen B. (2018). Deep learning approach for classification of breast cancer. Proceedings of the 2018 International Conference on Artificial Intelligence and Data Processing (IDAP).

[21] LeCun Y., Bengio Y., Hinton G. (2015). Deep learning. Nature.

[22] Selvathi D., Poornila A.A. (2018). Deep learning techniques for breast cancer detection using medical image analysis. Biologically Rationalized Computing Techniques for Image Processing Applications.

[23] Munir K., Elahi H., Ayub A., Frezza F., Rizzi A. (2019). Cancer diagnosis using deep learning: A bibliographic review. Cancers.

[24] Wu B., Kausar T., Xiao Q., Wang M., Wang W., Fan B., Sun D. (2017). FF-CNN: An efficient deep neural network for mitosis detection in breast cancer histological images. Proceedings of the Annual Conference on Medical Image Understanding and Analysis.

[25] Gao F., Wu T., Li J., Zheng B., Ruan L., Shang D., Patel B. (2018). SD-CNN: A shallow-deep CNN for improved breast cancer diagnosis. Comput. Med. Imaging Graph..

[26] Wang Y., Sun L., Ma K., Fang J. (2018). Breast cancer microscope image classification based on CNN with image deformation. Proceedings of the International Conference Image Analysis and Recognition.

[27] Wang Z., Li M., Wang H., Jiang H., Yao Y., Zhang H., Xin J. (2019). Breast cancer detection using extreme learning machine based on feature fusion with CNN deep features. IEEE Access.

[28] Duran-Lopez L., Dominguez-Morales J.P., Conde-Martin A.F., Vicente-Diaz S., Linares-Barranco A. (2020). PROMETEO: A CNN-Based Computer-Aided Diagnosis System for WSI Prostate Cancer Detection. IEEE Access.

[29] Chiang J.H., Chao S.Y. (2007). Modeling human cancer-related regulatory modules by GA-RNN hybrid algorithms. BMC Bioinform..

[30] Moitra D., Mandal R.K. (2019). Automated AJCC staging of non-small cell lung cancer (NSCLC) using deep convolutional neural network (CNN) and recurrent neural network (RNN). Health Inf. Sci. Syst..

[31] Lane N., Kahanda I. (2020). DeepACPpred: A Novel Hybrid CNN-RNN Architecture for Predicting Anti-Cancer Peptides. Proceedings of the International Conference on Practical Applications of Computational Biology & Bioinformatics.

[32] Bronstein M.M., Bruna J., LeCun Y., Szlam A., Vandergheynst P. (2017). Geometric deep learning: Going beyond euclidean data. IEEE Signal Process. Mag..

[33] Kipf T.N., Welling M. (2016). Semi-supervised classification with graph convolutional networks. arXiv.

[34] Veličković P., Cucurull G., Casanova A., Romero A., Lio P., Bengio Y. (2017). Graph attention networks. arXiv.

[35] Cho K., Van Merriënboer B., Gulcehre C., Bahdanau D., Bougares F., Schwenk H., Bengio Y. (2014). Learning phrase representations using RNN encoder-decoder for statistical machine translation. arXiv.

[36] Lu M., Fan Z., Xu B., Chen L., Zheng X., Li J., Znati T., Mi Q., Jiang J. (2020). Using machine learning to predict ovarian cancer. Int. J. Med. Inform..

[37] Arora M., Dhawan S., Singh K. (2020). Data Driven Prognosis of Cervical Cancer Using Class Balancing and Machine Learning Techniques. EAI Endorsed Trans. Energy Web.

[38] Chiu H.J., Li T.H.S., Kuo P.H. (2020). Breast Cancer–Detection System Using PCA, Multilayer Perceptron, Transfer Learning, and Support Vector Machine. IEEE Access.

[39] Montelongo González E.E., Reyes Ortiz J.A., González Beltrán B.A. (2020). Machine Learning Models for Cancer Type Classification with Unstructured Data. Computación y Sistemas.

[40] Shiqi L., Jun Z., Shuxun W. Research on Colorectal Cancer Prediction and Survival Analysis with Data Fusion Based on Deep Learning. Proceedings of the 9th International Workshop on Computer Science and Engineering (WCSE 2019).

[41] Zhou Y., Graham S., Alemi Koohbanani N., Shaban M., Heng P.A., Rajpoot N. Cgc-net: Cell graph convolutional network for grading of colorectal cancer histology images. Proceedings of the IEEE/CVF International Conference on Computer Vision Workshops.

[42] Schulte-Sasse R., Budach S., Hnisz D., Marsico A. (2019). Graph Convolutional networks improve the prediction of cancer driver genes. Proceedings of the International Conference on Artificial Neural Networks.

[43] Wang C., Guo J., Zhao N., Liu Y., Liu X., Liu G., Guo M. (2019). A Cancer Survival Prediction Method Based on Graph Convolutional Network. IEEE Trans. Nanobiosci..

[44] Ramirez R., Chiu Y.C., Hererra A., Mostavi M., Ramirez J., Chen Y., Huang Y., Jin Y.F. (2020). Classification of Cancer Types Using Graph Convolutional Neural Networks. Front. Phys..

[45] Guyon I., Weston J., Barnhill S., Vapnik V. (2002). Gene selection for cancer classification using support vector machines. Mach. Learn..

[46] Vaswani A., Shazeer N., Parmar N., Uszkoreit J., Jones L., Gomez A.N., Kaiser L., Polosukhin I. (2017). Attention is all you need. arXiv.

